# CRMP2 and voltage-gated ion channels: potential roles in neuropathic pain

**DOI:** 10.1042/NS20170220

**Published:** 2018-03-30

**Authors:** Lindsey A. Chew, Rajesh Khanna

**Affiliations:** 1 Department of Pharmacology, College of Medicine, University of Arizona, Tucson, AZ 85724, U.S.A.; 2 Department of Anesthesiology, College of Medicine, University of Arizona, Tucson, AZ 85724, U.S.A.; 3 Department of Neuroscience Graduate Interdisciplinary Program, College of Medicine, University of Arizona, Tucson, AZ 85724, U.S.A.; 4 The Center for Innovation in Brain Sciences, The University of Arizona Health Sciences, Tucson, AZ, U.S.A.

**Keywords:** CRMP2, Cav2.2, Nav1.7, Neuropathic pain, Trafficking

## Abstract

Neuropathic pain represents a significant and mounting burden on patients and society at large. Management of neuropathic pain, however, is both intricate and challenging, exacerbated by the limited quantity and quality of clinically available treatments. On this stage, dysfunctional voltage-gated ion channels, especially the presynaptic N-type voltage-gated calcium channel (VGCC) (Cav2.2) and the tetrodotoxin-sensitive voltage-gated sodium channel (VGSC) (Nav1.7), underlie the pathophysiology of neuropathic pain and serve as high profile therapeutic targets. Indirect regulation of these channels holds promise for the treatment of neuropathic pain. In this review, we focus on collapsin response mediator protein 2 (CRMP2), a protein with emergent roles in voltage-gated ion channel trafficking and discuss the therapeutic potential of targetting this protein.

## Introduction

Pain is universal and ravages effects across the globe without discriminating by age or gender [1]. Reported global sales for analgesics recently surpassed $22 billion, with purchases in the United States alone accounting for $13 billion of that overwhelming total [2]. Existing treatments, however, are inadequate and possess innumerable adverse side effects [3,4]. These therapies are further compromised by limited clinical efficacy and inflexibility across variable pain conditions. For this reason, new paradigms in developing treatments for pain are needed. Here, we focus on collapsin response mediator protein 2 (CRMP2) as a novel therapeutic target uniquely positioned to combat pain via regulation of voltage-gated ion channels.

Amongst various painful neuropathies, hyperexcitability of peripheral sensory neurones and dorsal horn spinal cord neurones represent commonly shared traits [5–7]. The presence of mushroom synapses in these circuits increases, with facilitation of excitatory synaptic transmission and depression of inhibitory input accompanying dendritic spines’ morphological changes to augment pain signal propagation [7,8]. Voltage-gated calcium channels (VGCCs) in these neuronal populations further influence nociceptive signaling by tuning neurotransmitter release and mediating membrane depolarization-dependent intracellular signaling via calcium ions (Ca^2+^) [9–11]. Notably, opioid receptor-targetted treatments also provide pain relief through downstream modulation of VGCCs [12]. Dysfunction and dysregulation of voltage-gated sodium channels (VGSCs) – namely increased sodium channel activity, current density, and negatively shifted half maximal activation voltage – also contribute to reduced thresholds for action potential initiation and heightened spike firing underlying the pathophysiology of pain [6]. These features highlight the position of primary afferent and second order neurones in neuropathic pain signaling pathways, while simultaneously underscoring the utility of therapeutic approaches targetting VGCCs and VGSCs.

Although several existing pain treatments directly block VGCCs or VGSCs, adverse side effects (such as disruption of normal physiological function in both the central and peripheral nervous systems) limit these drugs’ therapeutic use. Prialt (synthetic ω-conotoxin, also called Ziconotide) is one example of an N-type Ca^2+^ channel (Cav2.2) specific blocker that has been FDA-approved for clinical treatment of severe pain. However, Ziconotide also inhibits sympathetic norepinephrine release, posing a threat to normal cardiovascular physiology, and so it must be restricted to a narrow therapeutic window and intrathecal route of administration [13,14]. Obstacles have plagued the development of VGSC inhibitors, ranging from non-specificity to blood–brain barrier impermeability, as well. A structurally novel benzodiazepine-based state-dependent Nav1.7 blocker, for example exhibited poor pharmacokinetic properties and inefficacy against inflammatory pain states despite intravenous or intrathecal routes of administration [15–17]. While several other Nav1.7 inhibitors demonstrated high target affinity, low nanomolar affinity for other VGSC isoforms has been an insurmountable hurdle to date [18–20]. Despite these impediments, Ziconotide’s parameters of use and clinical indications validate presynaptic Cav2.2 at the spinal synapse, between primary sensory and spinal cord neurones, as a target for chronic pain. Similarly, the benzodiazepine-based inhibitor’s successful elimination of spontaneous neural firing and reversal of mechanical allodynia, in an experimental rodent model of spinal nerve ligation (SNL)-induced neuropathic pain, reassert the value of Nav1.7 as a therapeutic target [16,17].

Indirectly targetting voltage-gated ion channels through their interacting protein partners represents an emerging approach in drug discovery for neuropathic pain. Recent studies have identified CRMP2 as a part of the nociceptor transcriptome and uncovered CRMP2’s novel interactions with select VGCCs and VGSCs [21–32]. While CRMP2 traditionally promotes neurite outgrowth, neuronal polarization, progenitor proliferation, radial migration and assembly of microtubule networks, interactions along the CRMP2–Cav2.2 and CRMP2–Nav1.7 axes have been increasingly acknowledged and investigated as critical mechanisms mediating pain signals [33–39]. Two variants of CRMP2 exist: a long (~68–75 kDa) and short (~62 kDa) form. Both variants share a common core polypeptide, but divergent N-terminal domains result from alternative mRNA splicing [41]. Here, we focus on the short form, which localizes in both axons and dendrites, while the long form is primarily expressed in neuronal cell bodies [41]. To promote microtubule assembly and neurite outgrowth, CRMP2 enhances tubulin GTPase activity to facilitate tubulin polymerization [40–42]. Interestingly, CRMP2 phosphorylation by cyclin dependent kinase-5 (Cdk5) at Ser^522^ arrests axonal growth, and expression of both CRMP2 and Cdk5 increase in neuropathic pain states [43–48]. Work demonstrating the capacity of Cdk5-inhibiting compounds, such as roscovitine, to alleviate neuropathic and inflammatory pain provide a link between post-translational modifications of CRMP2 and pain phenotypes [49–51].

**Figure 1:**
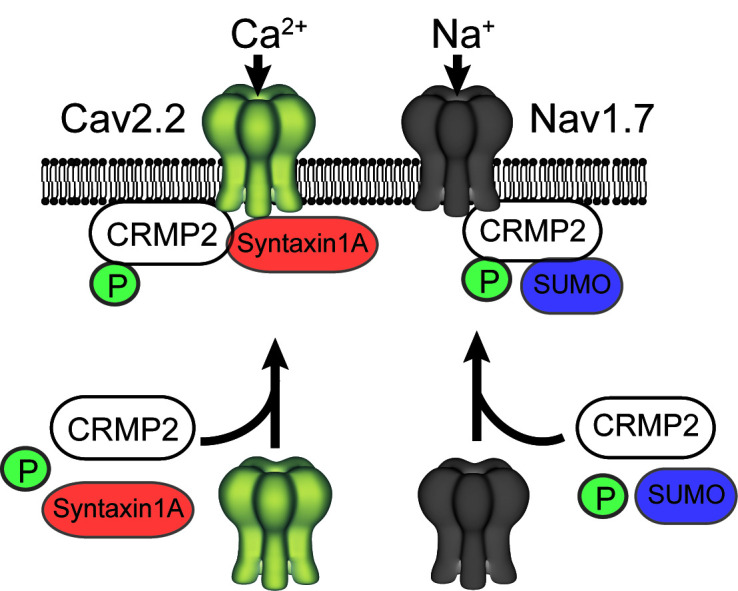
CRMP2 and ion channel signaling Cav2.2 and Nav1.7 channels exist in at least two pools – membrane-delimited and cytosolic. Cdk5-mediated phosphorylation of CRMP2 (at Ser^522^) and CRMP2-binding with syntaxin 1A facilitate the CRMP2–Cav2.2 interaction and increased Cav2.2 membrane insertion. In a parallel process, CRMP2 phosphorylation by Cdk5 supports CRMP2 SUMOylation (at Lys^374^) and subsequent increased Nav1.7 trafficking and surface expression.

In this review, we explore the mechanisms underlying CRMP2’s regulation of VGCC and VGSC surface expression. We also begin to unravel the code of post-translational modifications directing CRMP2 function, especially phosphorylation and SUMOylation (Small Ubiquitin-Like Modifier (SUMO)). By considering contributions of other CRMP2 binding partners, we investigate yet another layer of CRMP2’s exquisite regulation of voltage-gated ion channel trafficking. Finally, we examine the potential of these mechanisms as therapeutic targets for neuropathic pain and implications for on-going drug discovery ventures (Figure 1).

## Dysregulation of VGCCs in neuropathic pain

In neurones, Cav2.2 mediates Ca^2+^ influx as a primary precursor event triggering synaptic vesicle docking [51–55]. Interactions between Cav2.2 and the synaptic protein syntaxin 1A then follow to produce fast, synchronous neurotransmitter release [56–60]. Although multiple naturally occurring splice variants of Cav2.2 exist, a subset of splice forms (i.e. exons e37a and e37b) is highly expressed in presynaptic terminals; notably, neuropathic pain states up-regulate expression of these variants, especially in small diameter nociceptors of dorsal root ganglia (DRG) and within laminae I/II of the dorsal horn of the spinal cord [61,62]. Observations also show that Cav2.2 knockout mice lack typical responses to noxious stimuli, emphasizing these channels’ importance for thermal and mechanical nociception [63–70].

Yet, previous strategies directly blocking the pore-forming α subunit of Cav2.2 yielded limited success, and side effects of Ziconotide, for example ranged from confusion, depression, and hallucinations to decreased alertness, somnolence, orthostatic hypotension, and nausea [71–73]. Gabapentin, which targets the α2δ1 auxiliary subunit of Cav2.2 and dampens α2δ1-dependent VGCC membrane insertion, utilizes an indirect approach and possesses clinical indications for diabetic neuropathy, neuropathic pain, trigeminal neuralgia, and fibromyalgia [74–84]. Recently reported competition between the KCa1.1 (BK) potassium channel’s N-terminus and Cav2.2’s pore-forming α subunit for binding to the α2δ subunit further validates α2δ-targetting for analgesic strategies to combat neuropathic and inflammatory pain [85]. However, long-term use of gabapentin is restricted by evidence for its interference with synaptogenesis, and therefore memory formation, through disruption of α2δ1–thrombospondin interactions [86]. These shortcomings pair with Ziconotide and gabapentin’s state-independent channel blocking properties to mandate extra caution in their clinical applications. Efforts to harness state-dependent mechanisms for modulating Cav2.2 activity have been less risky, cumulating in the development of state-dependent, small molecule inhibitors such as TROX-1 [87,88]. Despite early successes in producing analgesic effects, TROX-1’s off-target interactions with Cav2.1 (P/Q-type channels) and Cav2.3 (R-type channels), combined with induction of mild locomotor and cardiovascular impairment at extremely high doses, have hindered its advancement [87].

### Interfering with CRMP2’s calcium channel-binding domain

Indirectly targetting Cav2.2 via its interactions with the adaptor protein CRMP2, however, has proven more fruitful [22–32]. A proteomic screen of Cav2.2 interacting partners identified CRMP2, subsequently, multiple direct CRMP2–Cav2.2 binding interfaces (i.e. several 15-amino acid long CaV-binding domains (CBD1–CBD3)) were discovered using peptide arrays [22,27]. In a series of studies examining targets of synthetic CBD peptides, conjugated to the HIV1 TAT (t-) domain for cell penetrance, only t-CBD3, comprising CRMP2 residues 484–498, succeeded in biochemically inhibiting the CRMP2–Cav2.2 interaction [27]. t-CBD3 also reduced Ca^2+^ currents in rat DRG sensory neurones and excitatory synaptic transmission in lamina II neurones from spinal cord slices [26]. Presence of t-CBD3 significantly inhibited the frequency of spontaneous excitatory postsynaptic currents (sEPSCs) without altering their amplitude, strongly suggesting that t-CBD3, and therefore CRMP2, functions via presynaptic rather than post-synaptic mechanisms of neurotransmission, likely to be mediated by Cav2.2 [27]. Since CRMP2 facilitates synaptic vesicle loading in hippocampal neurones, inhibition of the CRMP2–Cav2.2 complex would predictably reduce the recruitment of synaptic vesicles to Cav2.2 localized in the membrane [24]. It follows that knockdown of CRMP2 reduced evoked release of the pro-nociceptive neuropeptide calcitonin gene-related peptide (CGRP) in rat DRGs as well [25]. In a parallel strategy, t-CBD3 reduced evoked CGRP release alongside CGRP-dependent dilation of dural blood vessels in rodents; these data provide molecular and cellular underpinnings for t-CBD3’s ability to suppress tactile hypersensitivity in rodent models of HIV treatment-induced peripheral neuropathy and formalin-induced inflammatory pain [27]. Subsequent studies documented t-CBD3-mediated disruption of the CRMP2–Cav2.2 complex and efficacy in migraine, AIDS therapy-induced peripheral neuropathy, and tibial nerve injury-induced neuropathic pain [23,89,90] (Table 1).

**Table 1:** Biologics, drugs, and genetic strategies targetting CRMP2 in neuropathic pain

	Neuropathic pain models	Molecular effects	References
**Peptides**			
t-CBD3	• Reverses mechanical allodynia in:	• Inhibits CRMP2-Cav2.2 interaction	[27,89]
	- AIDS therapy (ddC)-induced neuropathic pain	• Reduces Ca^2+^ currents and neuronal excitability in DRGs, sEPSCs in lamina II spinal cord slices, evoked CGRP release, and capsaicin- induced meningeal blood flow	
	- 2.5% Formalin-induced pain (but not edema)		
	• Reverses capsaicin-induced nocifensive behavior		
	• Does not impair locomotion and spatial memory retrieval or induce depression/despair-associated behavior		
t-CBD3-G14F	• Reverses mechanical allodynia in:	• Reduces capsaicin-stimulated meningeal blood flow	[23]
	- AIDS therapy (d4T, stavudine)-induced neuropathic pain	• Targets trigeminal ganglion neurones	
t-CBD3-A6K	• Reverses mechanical allodynia in:	• Increases disruption of CRMP2–Cav2.2 interaction (than t-CBD3)	[89]
	- d4T-induced neuropathic pain	• Greater conformational stability (than t-CBD3)	
		• Reduces neuronal excitability, T-type/R-type Ca^2+^ currents, and evoked CGRP release in DRGs	
		• Reverses mechanical allodynia in d4T-induced neuropathic pain	
R9-CBD3	• Reverses mechanical allodynia in:	• Inhibits CRMP2–Cav2.2 interaction	[91,93]
	- tibial nerve injury-induced neuropathic pain	• Reduces depolarization-evoked Ca^2+^ influx in DRGs	
	- ddC-induced neuropathic pain		
myr-t-CBD3	• Reverses thermal hyperalgesia in:	• Higher peptide retention in cellular targets (than t-CBD3)	[94]
	- carrageenan-induced inflammatory pain	• Biochemically inhibits CRMP2–Cav2.2 interaction	
	- paw incision-induced post-surgical pain	• Reduces DRG excitability	
	• Reverses mechanical allodynia in:	• Inhibits Cav2.2 membrane localization, depolarization-evoked Ca^2+^ influx, and Ca^2+^ currents	
	- paw incision-induced post-surgical pain		
	• Does not impair locomotion, induce paralysis, or cause conditioned place preference for a peptide-paired		
R9-CBD3-A6K	• Reversibly attenuates mechanical allodynia in:	• Inhibits CRMP2–Cav2.2 interaction and Cav2.2 membrane localization	[90]
	- tibial nerve injury-induced neuropathic pain	• Reduces depolarization-evoked Ca^2+^ influx in DRGs	
	• Does not induce conditioned place preference with a peptide-paired chamber; impair locomotion or learning/recognition memory; induce anxiolytic or depression/despair	• Reduces neuronal excitability and T-type/R-type Ca^2+^ currents in DRGs	
		• Induces dopamine release in the nucleus accumbens (in pain states only)	
t-N-terminal fragment-CBD3	• Reverses mechanical allodynia and thermal hyperalgesia in:	• Inhibits CRMP2–Cav2.2 interaction, depolarization-evoked Ca^2+^ influx, and Ca^2+^ currents in DRGs	[92]
	- paw incision-induced post-surgical pain		
	- HIV-induced neuropathic pain		
t-CNRP1	• Reverses thermal hyperalgesia in:	• Inhibits CRMP2-syntaxin 1A and CRMP2–neurofibromin interactions	[115]
	- paw incision-induced post-surgical pain	• Reduces evoked CGRP release in spinal cord	
	- carrageenan-induced inflammatory pain (but not edema)	• Inhibits Cav2.2 membrane localization, depolarization-evoked Ca^2+^ influx, and Ca^2+^ currents in DRGs	
	• Reverses mechanical allodynia in:		
	- paw incision-induced post-surgical pain		
	- HIV-induced neuropathic pain		
**Small molecules**			
(*S*)-Lacosamide	• Reverses mechanical allodynia and thermal hyperalgesia in:	• Inhibits the CRMP2-Cav2.2 interaction and depolarization-evoked Cav2.2-mediated Ca^2+^ influx in both DRGs and cortical neurones	[101,103,115]
	- paw incision-induced post-surgical pain	• Directly inhibits Cdk5-mediated CRMP2 phosphorylation in DRGs	
	- SNL-induced neuropathic pain	• Inhibits Cav2.2 and Nav1.7 surface expression and Ca^2+^ currents	
	- SNI-induced neuropathic pain	• Reverses CRISPR/Cas9-editing of *Nf1* induced hyperexcitability (i.e. increased action potential frequency, reduced rheobase) and changes in Nav1.7/Cav2.2-mediated current density	
	- CRISPR/Cas9-induced neurofibromin truncation (missing C-terminal domain)		
	• Does not impair locomotion		
**Genetic methods**			
CRMP2 overexpression		In hippocampal neurones:	[24,25]
		• Increases surface Cav2.2 expression and Ca^2+^ currents	
		• Increases synaptic vesicle recycling	
		• Enhances glutamate release	
		• Increases numbers of synaptic boutons	
		In DRGs:	
		• Increases surface Cav2.2 expression and Ca^2+^ currents	
CRMP2 shRNA/siRNA/CRISPR	• Reverses thermal hyperalgesia in:	• Inhibits Ca^2+^ currents in hippocampal neurones and DRGs	[24,25,116]
	- t-RFP-Nf1-Cas9 lentivirus-infected rats (missing C-terminal domain of neurofibromin)	• Reduces Cav2.2 surface expression, τ_activation_ for Ca^2+^ currents, and evoked CGRP release in DRGs	
		• Inhibits Na^+^ currents in DRGs from virus-infected rats	
CBD3 AAV vector	• Reverses mechanical allodynia, thermal hyperalgesia, and cold thermal hypersensitivity in:	• Inhibits N-type and T-type Ca^2+^ currents in DRGs from rats injected with AAV-CBD3	[97]
	- SNI-induced neuropathic pain		

Abbreviations: AAV, adeno-associated viral; CDB3; calcium binding domain 3; CGRP, calcitonin gene-related peptide; CNRP1, CRMP2–neurofibromin regulating peptide; CRISPR, clustered regularly interspaced short palindromic repeats; d4t, 2′,3′-didehydro-2′,3′-dideoxythymidine (Stavudine); ddC, 2’,3’ dideoxycytidine; sEPSC, spontaneous excitatory postsynaptic current; SNI, spared nerve injury.

Importantly, disrupting the CRMP2–Cav2.2 interaction did not alter core sympathetic nervous system functions such as pulsatile arterial pressure, mean arterial pressure, heart rate, and core body temperature [91]. Several evolutionary progressions of the CBD3 peptide were synthesized, with each mutation of the original peptide sequence intended to improve its cell penetrance and structural stability [23,90–94]. Antinociceptive effects of one such peptide were reversible in a rodent model of neuropathic pain, and nociceptive behaviors readily resurfaced once peptide administration ceased [90]. Continuous peptide administration did not result in tolerance to its analgesic properties [90]. Although addiction represents a major hazard of currently available pain therapeutics, CBD3 peptides did not cause conditioned place preference for a peptide administration paired chamber and increased dopamine release in the nucleus accumbens of injured, but not naïve, rodents [90]. These results suggest that uncoupling the CRMP2–Cav2.2 complex is non-addictive, while still engaging the centralized affective component of pain and peripheral sensory mechanisms [95,96]. A battery of assessments for anxiety-, despair/depression-, locomotion-, and cognition/recognition memory-associated effects (i.e. light-dark box, elevated plus maze, open field, tail suspension, rotorod, and novel object tests) also found no significant difference in the behaviors of peptide-treated rodents compared with vehicle-treated counterparts [27,90]. Centralized off-target side effects of CBD3 peptides are unlikely given the wide array of tests demonstrating normal behavior in tasks requiring core central nervous system participation. Providing additional validation for this therapeutic strategy, adenoviral injection of CBD3 into rodents’ lumbar DRGs afforded protection from spared nerve injury (SNI)-induced neuropathic pain for over 6 weeks [97].

Investigating CBD3’s pharmacological mechanism by synthesizing Cav2.2-derived peptides revealed that Cav2.2’s first intracellular loop (L1-Cav2.2, residues 388–402) and distal C-terminus (Ct-Cav2.2, residues 2014–2028) directly bind CRMP2 [97]. Binding affinity of Ct-Cav2.2/CRMP2 was ~75-fold greater than between L1-Cav2.2/CRMP2, and only disruption of the Ct-Cav2.2/CRMP2 interaction inhibited evoked CGRP release in rat DRGs [98]. Notably, there is limited distal Ct homology amongst VGCCs, while sequence conservation of L1 is greater than 60%. In a complementary study to identify CRMP2’s key residues mediating the Cav2.2 interaction, the CBD3 peptide was divided into shorter fragments: the first six amino acids (N-terminal fragment) and the last nine (C-terminal fragment) [93]. In rat DRGs, only CBD3’s N-terminal fragment successfully inhibited depolarization-evoked calcium influx, reduced Ca^2+^ currents, and disrupted the CRMP2–Cav2.2 interaction *in situ* [93](Table 1). Through selective single site mutagenesis, further optimization of the N-terminal fragment and *in vivo* assessments identified three key features facilitating disruption of this interaction by a CBD-derived peptide: (i) a positively charged site corresponding (guanidine group in arginine) at position 4 of the six-mers, (ii) a hydrophobic region (arginine residue) at position 4 of the six-mers; and (iii) a hydrogen bond acceptor (alanine) at the first position within the N-terminal fragment [93]. This discovery establishes three basic pharmacophore elements upon which biochemically and functionally similar small molecules can be designed, harnessing CRMP2’s regulation of Cav2.2 to tackle neuropathic pain.

### Cdk5-mediated phosphorylation of CRMP2 regulates Cav2.2 trafficking

The link between phosphorylation of CRMP2 by Cdk5 and pain phenotypes warranted further exploration. Knowledge that Cdk5-mediated phosphorylation of CRMP2 arrests axonal growth encouraged the search for a molecule capable of inhibiting CRMP2-dependent neurite outgrowth. The antiepileptic drug (2R)-2-(acetylamino)-N-benzyl-3-methoxypropanamide ((*R*)-LCM, tradename Vimpat®) fits this profile, and subsequent characterization of (*R*)-LCM’s molecular derivatives uncovered (*S*)-Lacosamide ((*S*)-LCM), which also targets CRMP2 [43–45,99,100]. (*S*)-LCM selectively inhibits Cdk5-mediated phosphorylation of CRMP2, and a combination of structural, biophysical studies and *in silico* docking models contributed to the determination of (*S*)-LCM’s precise binding pocket [101]. In parallel, it was discovered that phosphorylation of CRMP2 by Cdk5 dynamically regulates and increases the association of CRMP2 with Cav2.2 [102]. While activation of Cdk5 and increased CRMP2 expression in rat DRGs contribute to several pain phenotypes, (*S*)-LCM biochemically disrupts the CRMP2–Cav2.2 interaction while inhibiting depolarization-evoked Ca^2+^ influx and Ca^2+^ currents [46,49,102,103]. Systemic administration of (*S*)-LCM to mice, at three daily doses of 20 mg/kg over 4 days, successfully uncoupled the CRMP2–Cav2.2 interaction in brain lysates, with no detectable effects on locomotion, feeding, or behavior [103]. In experimental rodent models of SNL- and SNI-induced neuropathic pain, (*S*)-LCM administration effectively reversed nociceptive behaviors within 30 and 120 min, respectively [103]. These findings add the inhibition of Cdk5-mediated phosphorylation of CRMP2 as a potential therapeutic strategy for eliminating chronic pain.

### Unraveling a larger CRMP2 complex: neurofibromin and syntaxin 1a

Studies centered on dysregulation of neurofibromin, a tumor suppressor gene product, have also demonstrated that CRMP2 interacts with neurofibromin’s C-terminal domain, and that the two proteins co-localize in neurites [104,105]. Strikingly, neurofibromatosis type-1 (NF1) patients, in whom neurofibromin is often mutated or truncated, experience idiopathic chronic pain for which opioids often fail to provide relief [106–109]. Observations of DRGs in *Nf1*^+/−^ haploinsufficient mice revealed augmented sodium (Na^+^) current densities (both tetrodotoxin (TTX)-sensitive and TTX-resistant), alongside increased N-type Ca^2+^ currents and enhanced stimulus-evoked release of glutamate, substance P, and CGRP [110–113]. Interestingly, treatment of *Nf1*^+/−^ DRGs with the aforementioned t-CBD3 inhibited both Cav2.2-mediated currents and CGRP release [98]. These results not only link ion channel dysregulation, but also CRMP2, to the pathophysiology of NF1, and suggest that sensitization of small diameter nociceptive sensory neurones may explain the pain reported by NF1 patients [114].

To investigate the physiological role of the CRMP2–neurofibromin interaction, a CRISPR/Cas9 strategy truncating the C-terminal domain of neurofibromin was adopted and then DRGs from these CRISPR/Cas9-edited rats were electrophysiologically characterized [115]. These recordings demonstrated that CRISPR/Cas9-editing of *Nf1* and the resulting neurofibromin truncation in rat DRGs caused a remodeling of ion channels with biophysical properties essentially parallel to those of *Nf1*^+/−^ DRGs; augmented Na^+^ and Ca^2+^ currents were observed in addition to hyperexcitability (determined by increased action potential frequency and decreased threshold), while K^+^ currents were not significantly different [112]. In both male and female rats, neurofibromin truncation led to the development of thermal hyperalgesia [115], dispeling previous reports of possible sexual dimorphism in the behavioral responses.

Biochemical examination of DRGs showed that neurofibromin truncation permitted an up-regulation in phosphorylation of CRMP2 by Cdk5 [115]. Given the previous identification of (*S*)-LCM as a selective inhibitor of Cdk5-mediated phosphorylation of CRMP2, DRGs, with neurofibromin truncation were treated with (*S*)-LCM; this inhibited membrane localization of Cav2.2 and normalized the previously detected changes in both Na^+^ and Ca^2+^ currents as well as action potential frequency, threshold, and overall hyperexcitability [115]. Oral administration of (*S*)-LCM to rats previously subjected to CRISPR/Cas9-editing of *Nf1* reduced thermal hyperalgesia in both males and females [115]. A subsequent study demonstrated that lentiviral knockdown of CRMP2 expression in rodents subjected to neurofibromin truncation was sufficient to attenuate thermal hyperalgesia and normalize Na^+^ current density [116]. Although the precise nature of NF1-linked pain syndromes remains unknown, patients clearly experience pain independent of their tumor burden, and these findings affirm the necessity of CRMP2 in NF1-related pain, while suggesting a physiological role for neurofibromin’s C-terminal domain-mediated inhibition of the CRMP2–Cav2.2 interaction [106].

Efforts to fully understand neurofibromin-mediated regulation of CRMP2, and its consequences for ion channel activity, led to the synthesis of complementary peptide arrays on which CRMP2 and neurofibromin’s C-terminus were tiled, respectively [117]. Multiple binding domains were identified, and corresponding peptides were synthesized – including a unique CRMP2–neurofibromin regulating peptide (CNRP1) that, when conjugated to tat (t-), disrupts the CRMP2–neurofibromin interaction while also inhibiting membrane localization of Cav2.2, depolarization-evoked Ca^2+^ influx, and Ca^2+^ currents in rat DRGs [117]. To test the hypothesis that neurofibromin prevents CRMP2’s association with additional protein partners engaged in Cav2.2 trafficking, CRMP2 binding interactions within a nanodisc-embedded synaptic membrane library (which preserved membrane protein integrity in a virtually native environment without use of detergents) were assessed [117]. MS of co-immunoprecipitated CRMP2-bound proteins, in the presence and absence of t-CNRP1, identified syntaxin 1A as a novel CRMP2 binding partner (at residues 456–480) [117].

Syntaxin 1A is widely acknowledged as a regulator of Cav2.2 and Cav2.2-associated neurotransmitter release [54,58,118]. Recruitment of syntaxin 1A to Cav2.2’s intracellular SYNaptic Protein INTeraction (‘synprint’) region facilitates trafficking of the channel and promotes synaptic vesicle docking [55,118–120]. Past efforts to harness uncoupling of the syntaxin 1A–Cav2.2 interaction for therapeutic use have not advanced despite an early report of success [121]. However, these recent discoveries implicating CRMP2 in neurotransmitter release may support reconsideration of this strategy.

Notably, syntaxin 1A binds CRMP2 in the same region as the C-terminal domain of neurofibromin [117]. Disrupting the CRMP2–syntaxin 1A interaction via t-CNRP1 inhibited evoked CGRP release in spinal cord, and intrathecal administration of t-CNRP1 alleviated nociceptive behaviors in carrageenan-induced inflammatory pain, post-surgical pain, and HIV-induced (gp120-evoked) sensory neuropathic pain [117]. This evidence suggests that neurofibromin plays a physiological role in sequestration of CRMP2 from syntaxin 1A to disrupt Cav2.2 trafficking, limit synaptic vesicle docking, mitigate CGRP release, and ultimately, control pain signaling. However, loss of neurofibromin, as observed in NF1 patients, results in disinhibition of the tripartite CRMP2–Cav2.2–syntaxin 1A interaction, therefore permitting increased N-type Ca^2+^ currents and CGRP release. Precedence for therapeutic targetting of synaptic vesicle proteins already exists (e.g. levetiracetam), and curbing the CRMP2–syntaxin 1A interaction may be especially advantageous in efforts to relieve not only NF1-related pain, but neuropathic pain at large [122].

### Synergistic regulation of T-type and R-type VGCCs in neuropathic pain

T-type (i.e. Cav3.1, Cav3.2, and Cav3.3) and R-type (i.e. Cav2.3) Ca^2+^ channels also represent acknowledged molecular targets for the development of pain therapeutics. In rat DRGs and dorsal horn spinal cord neurones, evidence suggests that both T-type and R-type Ca^2+^ currents contribute to neuronal excitability [123–126]. While fast kinetic properties and high open probabilities at only very negative membrane potentials characterize T-type Ca^2+^ channels, R-type Ca^2+^ channels exhibit slow kinetic properties and, in pyramidal hippocampal neurones, the capacity to modulate both after depolarization and burst firing [127–129]. Previous efforts to harness direct T-type Ca^2+^ channel blockers (e.g. mibefradil and ethosuximide) yielded mild success in relieving nociceptive behaviors in various neuropathic pain models; antisense oligonucleotide-mediated silencing of T-type channels attenuated nociception in a chronic constrictive injury model of neuropathic pain as well [130–135]. Obstacles posed by side effects and blood–brain barrier impermeability, however, have limited their advancement [130–132]. Likewise, impediments have faced R-type channel blockers – such as SNX-482, which demonstrated antinociceptive effects in several experimental chronic neuropathic pain models, but appears to incompletely block VGSCs in addition to L-type and P/Q-type Ca^2+^ channels [136–138].

Indirect T-type channel blocking strategies, however, have proven more fruitful and capable of overcoming the aforementioned hurdles. Ubiquitination of Cav3.2 within the III-IV linker, for example controls its surface expression; a homeostatic balance between ubiquitin ligase WWP1 and ubiquitin protease USP5 activity in turn regulate Cav3.2 activity [139]. Given this, one promising strategy disrupts the Cav3.2–USP5 interface with interfering peptides and small molecules that operate on principles similar to that of the CBD3 peptide and its derivatives [140,141]. Uncoupling the Cav3.2–USP5 interaction produces analgesia *in vivo*, and these advances pave the way for other conceptually similar approaches that focus on a divergent molecular target [140,141].

While it remains unclear whether CRMP2 directly interacts with T-type and R-type Ca^2+^ channels, previous reports revealed that the CRMP2-derived peptide t-CBD3 reduced T-type Ca^2+^ currents [89]. A subsequent iteration of the peptide (t-CBD3-A6K, in which the sixth position was mutated from alanine to lysine for improved structural stability) demonstrated similar competence while simultaneously inhibiting T-type and R-type Ca^2+^ currents [89]. Substitution of the TAT cell penetration motif for a homopolyarginine (R9) strategy (R9-CBD3-A6K) produced a peptide with equivalent functional efficacy and improved membrane permeability [90,93]. R9-CBD3-A6K robustly reversed nociceptive behaviors in an experimental neuropathic pain model without eliciting side effects associated with Ziconotide or TROX-1 [90]. These observations suggest that the CRMP2-derived peptide exploits synergistic inhibition of a broad spectrum of VGCCs to produce potent relief from various neuropathic pain states. The mechanism by which CRMP2 itself mediates these effects remains unknown and requires further investigation. It is clear, however, that CRMP2 lies at a focal point of molecular traffic and represents a central player in the co-ordination of VGCCs. As such, CRMP2 holds substantial value as a therapeutic target for neuropathic pain.

## Dysregulation of VGSCs in neuropathic pain

Participation of VGSCs fundamentally shapes the character of action potentials, and variable VGSC-isoform expression profiles impact spike frequency as well as threshold from neurone to neurone [142,143]. Studies integrating clinical evidence with molecular tools have linked numerous mutations in both TTX-sensitive channels (e.g. Nav1.6 and Nav1.7) and TTX-resistant channels (e.g. Nav1.8 and Nav1.9) to inherited neuropathic pain syndromes such as erythromelalgia, paroxysmal extreme pain disorder, and small fiber neuropathy [144–148]. In particular, reports highlighting Nav1.7 enrichment in nociceptors have fostered interest in Nav1.7 as a target in drug discovery for neuropathic pain [149]. Pain states associated with chemotherapy-induced peripheral neuropathy and diabetic neuropathy up-regulate Nav1.7 and Nav1.7-dependent hyperexcitability of primary afferent sensory neurones [6,150,151]. Combined with existing knowledge of VGSC-associated signaling and trafficking molecules, these observations provide a platform for initial investigations, especially through mechanisms interfering with VGSC trafficking and membrane localization. For example, expression of sodium channel β2 subunits, which promote surface expression of TTX-sensitive Na^+^ channels through interactions with cell adhesion molecules and cytoskeletal proteins, increase in neuropathic pain states [152,153]. However, β2 subunits associate with multiple VGSC isoforms [154,155]. Similarly, the E3 ubiquitin ligase Nedd4-2 (neural precursor cell expressed developmentally down-regulated protein 4) labels VGSCs for endocytosis, but its loss in neuropathic pain states augments both Nav1.7 and Nav1.8 currents [156,157]. While reducing VGSC surface expression through these mechanisms is unlikely to confer specificity, other trafficking-oriented approaches remain and may prove advantageous.

SUMO protein, for instance is known to regulate a variety of voltage-gated ion channels, including Nav1.2, Kv1.5, and Kv2.1, and their membrane insertion [158–160]. SUMOylation is the covalent addition of a ~12-kDa SUMO groups to the lysine residue of a SUMO-interaction motif (SIM, usually a large hydrophobic residue (ψ) before the modification site lysine and a negatively charged amino acid two residues downstream (ψ-K-X-E/D)) [161,162]. The E2 ubiquitin-like ligase and SUMO-conjugating enzyme Ubc9 facilitates addition of SUMO moieties, while SUMO/sentrin-specific peptidases (SENP) 1 and SENP2 enable reversal of SUMOylation by removing them [163,164]. Previous studies have implicated SUMOylation in pain states. The antidiabetic drug rosiglitazone (Avandia®) induces SUMOylation of a peroxisome proliferator-activated receptor to suppress macrophage infiltration and nociceptive behaviors [165]. In rheumatoid arthritis, intrinsically high SUMO-1 levels couple with histone SUMOylation-mediated decreases in SENP1 expression to produce inflammatory pain [166]. Interestingly, amongst TTX-sensitive Na^+^ channels, only Nav1.7 lacks a putative SUMOylation motif, and its trafficking mechanism had not been clearly delineated [167].

### CRMP2 regulates Nav1.7 trafficking

To determine whether SUMOylation of a Nav1.7-binding partner mediates its membrane insertion, sequences of potential Nav1.7 protein partners were scanned for possible SIMs. This spurred the identification of CRMP2’s SIM (KMD, residues 374–376) [28]. In rat DRGs, a SUMO-impaired CRMP2 mutant (i.e. K374A) exhibited decreased Nav1.7-mediated currents and decreased binding in co-immunoprecipitation with Nav1.7, while a biotinylation assay detected significantly reduced surface expression of Nav1.7 [28,29,169]. Notably, Nav1.1- and Nav1.3-mediated currents were unaffected by SUMO-impairment of CRMP2 [28]. Given that an equilibrium naturally exists between CRMP2’s tetrameric and monomeric states, Nav1.7 currents were assessed in CAD cells (derived from a catecholaminergic mouse cell line) transfected with a molar 3:1 ratio of wild-type CRMP2:CRMP2-K374A cDNAs [28,168]. Under these conditions, Nav1.7 currents were markedly reduced in comparison with CAD cells containing only wild-type CRMP2, suggesting that a single SUMO-impaired CRMP2 monomer is sufficient to impact Nav1.7 currents [28]. Although potential CRMP2 SIMs were identified at K20 and K390, mutations at these sites of CRMP2 (i.e. K20A and K390A) did not reduce Nav1.7 currents or Nav1.7 surface expressions [29]. These findings provided the first evidence for CRMP2’s role as a signaling molecule in Nav1.7 trafficking, where loss of CRMP2 SUMOylation drives dissociation of the Nav1.7–CRMP2 interaction.

Subsequent questions centered on regulation of Nav1.7 function by CRMP2’s hierarchical post-translational modification code, including SUMOylation and phosphorylation by Cdk5, Src family tyrosine-kinases Fyn and Yes, Rho-associated protein kinase (RhoK), and glycogen synthase kinase 3β (GSK3β). In rat DRGs, Nav1.7 membrane localization and current density were reduced by a p-null CRMP2 mutant inaccessible to Cdk5 (i.e. S522A), but not by those mutants inaccessible to other kinases (i.e. Fyn (Y32F), Yes (Y479F), GSK-3β (T509A/T514A), RhoK (T555A)) [169]. Notably, impairment of both CRMP2 SUMOylation and Cdk5-mediated phosphorylation (i.e. K374A/S522A) did not further reduce Nav1.7 currents or surface fraction from decreases independently imposed by either mutation [169]. In CAD cells, Cdk5 p-null CRMP2 was inaccessible to SUMOylation, but impairment of CRMP2 SUMOylation did not alter its Cdk5-mediated phosphorylation [163]. Rat DRGs expressing either SUMO-impaired or Cdk5 p-null CRMP2 also exhibited reduced excitability (i.e. decreased action potential frequency) [169]. However, simultaneous loss of CRMP2 SUMOylation and Fyn-mediated phosphorylation (i.e. K374A/Y32F) failed to reduce Nav1.7 currents and surface localization compared with wild-type CRMP2 conditions [169]. Total Nav1.7 expression did not change, and Nav1.1, Nav1.3, Nav1.5, Nav1.6, and TTX-resistant Na^+^ currents were not affected by impairment of these CRMP2 post-translational modifications [28,169]. Parallel study of Nav1.7 currents in human DRGs revealed congruent effects of SUMO-impaired and Cdk5 p-null CRMP2 mutants on both TTX-sensitive and total Na^+^ currents [169]. Together, these results convey that Cdk5-mediated phosphorylation is required for CRMP2 SUMOylation and its promotion of Nav1.7 function, while Fyn-mediated phosphorylation is an obligatory precursor to Nav1.7 internalization driven by CRMP2 deSUMOylation.

Previous reports have described Nedd4-2-mediated labeling of Nav1.7 for internalization, in addition to CRMP2-mediated endocytosis of L1-cell adhesion molecules through interactions with the endocytic protein Numb that recruits epidermal growth factor receptor pathway substrate 15 (Eps15) to induce membrane curvature and initiate clathrin-mediated endocytosis [156,157,170–173]. In rat DRGs expressing SUMO-impaired CRMP2, individually eliminating any of these internalization protein partners or pharmacological inhibition of clathrin-mediated endocytosis via Pitstop2, rescued loss of Nav1.7 current [169]. This implies that Numb, Eps15, and Nedd4-2 are requisite participants in Nav1.7’s clathrin-mediated internalization. By contrast, Cdk5-mediated phosphorylation of CRMP2 exhibited reduced interactions with Numb, Eps15, and Nedd4-2 [169]. Not surprisingly, CRMP2 does not bind Numb, Eps15, and Nedd4-2 under loss of Fyn-mediated phosphorylation, regardless of SUMOylation status [169]. This work more completely describes the molecular participants and hierarchy of CRMP2’s post-translational modifications engaged in Nav1.7 internalization. While it remains unclear how CRMP2 exclusively targets Nav1.7 and not other isoforms of VGSCs, examination of intracellular loop sequences unique to Nav1.7 (e.g. the N-terminus and loop 2 connecting the second and third transmembrane domain modules) may reveal additional answers. Regardless, the highlighted conservation of Nav1.7 trafficking mechanisms between rodent and human DRGs presents an ideal opportunity for therapeutic efforts that disrupt the CRMP2–Nav1.7 interaction to selectively impede CRMP2 SUMOylation and attenuate neuropathic pain.

### CRMP2: a novel therapeutic target for neuropathic pain

It is apparent that CRMP2 functions as a multidimensional co-ordinator of protein–protein interactions, particularly in mediating surface trafficking of voltage-gated ion channels. Competition between syntaxin 1A and neurofibromin for a singular CRMP2 binding domain regulates CRMP2’s direct interaction with Cav2.2. Cdk5-mediated phosphorylation further enhances the CRMP2–Cav2.2 association. This array of signaling interactions form an attractive target for therapeutic intervention against dysfunctional Cav2.2 in neuropathic pain. Given the potential multipharmacology of CRMP2-oriented strategies that reduce Cav2.2 activity, an obligation exists to explore CRMP2 interactions with T-type Ca^2+^ channels and R-type Ca^2+^ channels further. Expansion of the literature on CRMP2-Nav1.7 trafficking mechanisms also reinforces the importance of appreciating how subtle, yet fundamental, molecular events implicate protein in diverse biological roles. CRMP2’s hierarchical post-translational modification code yields immense potential for harnessing block of CRMP2 SUMOylation (as well as other modifications) to reduce Nav1.7 activity and alleviate neuropathic pain without disrupting core physiological functions. Importantly, CRMP2-derived peptides did not demonstrate toxicity or other adverse side effects associated with targetting VGCCs via direct and state-dependent channel blockers. CRMP2-targetted small molecules in development against VGSCs as well as VGCCs ([92,100,101,103,117], unpublished data) have also shown similar profiles of preclinical success. Together, this work precipitates the necessary advancement of CRMP2-centered drug discovery ventures into studies for diverse clinical pain indications and other channelopathies.

## Abbreviations

USP5: ubiquitin-specific protease 5
BK: big conductance calcium-activated potassium channel
CAD: Catecholaminergic A differentiated
Cdk5: cyclin dependent kinase-5
CGRP: calcitonin gene-related peptide
CNRP1: CRMP2–neurofibromin regulating peptide
CRISPR/Cas9: clustered regularly interspaced short palindromic repeats-associated protein 9- nuclease
CRMP2: collapsin response mediator protein 2
Ct-Cav2.2: distal C-terminus of Cav2.2
DRG: dorsal root ganglia
Eps15: epidermal growth factor receptor pathway substrate 15
FDA: Food and Drug Administration
(*S*)-LCM: (*S*)-Lacosamide
L1-Cav2.2: Cav2.2’s first intracellular loop
Nedd4-2: neural precursor cell expressed developmentally down-regulated protein 4
NF1: neurofibromatosis type-1
RhoK: Rho-associated protein kinase
SENP: SUMO/sentrin-specific peptidase
SIM: SUMO-interaction motif
SNI: spared nerve injury
SNL: spinal nerve ligation
SUMO: small ubiquitin-like modifier
TROX-1: N-triazole oxindole
TTX: tetrodotoxin
VGCC: voltage-gated calcium channel
VGSC: voltage-gated sodium channel
WWP1: NEDD4-like E3 ubiquitin-ligase
